# Data on minute DNA quantification on microvolumetric solutions: comparison of mathematical models and effect of some compounds on the DNA quantification accuracy

**DOI:** 10.1016/j.dib.2018.09.098

**Published:** 2018-10-02

**Authors:** Joana Carvalho, Renato Negrinho, Sarah Azinheiro, Alejandro Garrido-Maestu, Jorge Barros-Velázquez, Marta Prado

**Affiliations:** a International Iberian Nanotechnology Laboratory, Av. Mestre José Veiga s/n, 4715-330 Braga, Portugal; b Machine Learning Department, Carnegie Mellon University, Pittsburgh, PA 15213, USA; c Department of Analytical Chemistry, Nutrition and Food Science, School of Veterinary Sciences/College of Biotechnology, University of Santiago de Compostela, Campus Lugo, Lugo, Spain

## Abstract

This article contains data related to the research article entitled “Novel approach for accurate minute DNA quantification on microvolumetric solutions” (Carvalho et al., 2018). The combination of PicoGreen® with a microvolume fluorospectrometer is a popular DNA quantification method due to its high sensitivity and minimal consumption of sample, being commonly used to evaluate the performance of microfluidic devices designed for DNA purification. In this study, the authors present data related with the effect of DNA fragmentation level. The present data article includes the data used on the precision evaluation, in terms of repeatability, of the mathematical models developed to obtain the standards curve for salmon sperm DNA (low molecular weight). In addition, results related with the effect of some compounds on the DNA quantification accuracy using λDNA are presented.

**Specifications table**

**Table:** 

Subject area	Biology, Chemistry
More specific subject area	Molecular Biology, Analytical Chemistry
Type of data	Tables and figures
How data was acquired	The fluorescence signal of all the DNA samples was measured with the microvolume fluorospectrometer NanoDrop 3300 (Thermo Scientific™, Waltham, MA, US) using the PicoGreen® dye from Quant-iT^TM^ PicoGreen® dsDNA Assay kit (Molecular probes Inc., Eugene, USA).
Data format	Analyzed
Experimental factors	Not applicable
Experimental features	The DNA samples were mixed with the PicoGreen® working solution in a ratio 1:1 for a final volume of 20 µL. After 5 minutes, the fluorescence signal of each sample was obtained with NanoDrop 3300. The DNA quantification, in terms of DNA concentration, was performed using the equipment׳s software and three different mathematical models developed for comparison.
Data source location	Not applicable
Data accessibility	Data with this article
Related Research Article	J. Carvalho, R. Negrinho, S. Azinheiro, A. Garrido-Maestu, J. Barros-Velázquez, M. Prado, Novel approach for accurate minute DNA quantification on microvolumetric solutions, Microchem. J. (2018) 138, 540–549, https://doi.org/10.1016/j.microc.2018.02.001.

**Value of the data**

• The data presented here shows the effect of DNA fragmentation on the results of DNA quantification with PicoGreen® and NanoDrop 3300.
• Three mathematical models were used, adjusted and compared in terms of accuracy and precision for the quantification of fragmented DNA.
• We present as well data of the DNA quantification measurements using λDNA as standard, showing the influence of compounds commonly used in silica-based microscale Solid Phase Extraction (µSPE) methods for DNA purification.
• This data will help other researchers to evaluate their DNA quantification results and to choose the best adjustment depending on their type of sample.

## 1 Data

**Fig. 1:**
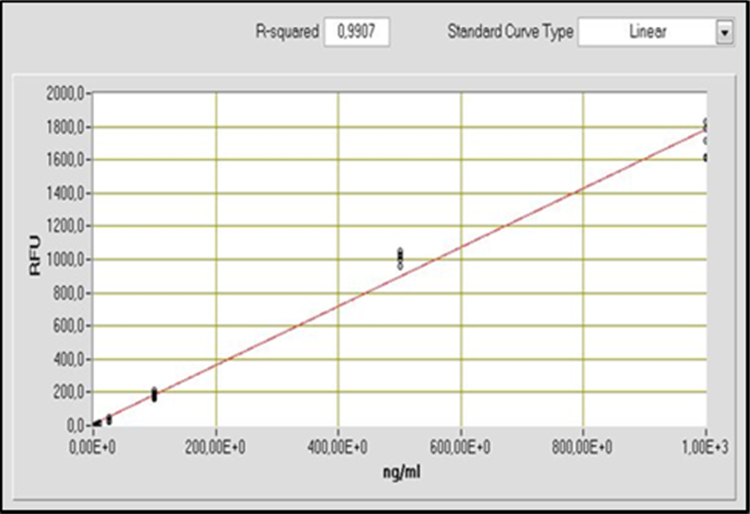
Mathematical adjustment of the standard curve data obtained for λDNA using NanoDrop׳s software: Linear adjustment with R-squared 0.9907.

**Fig. 2:**
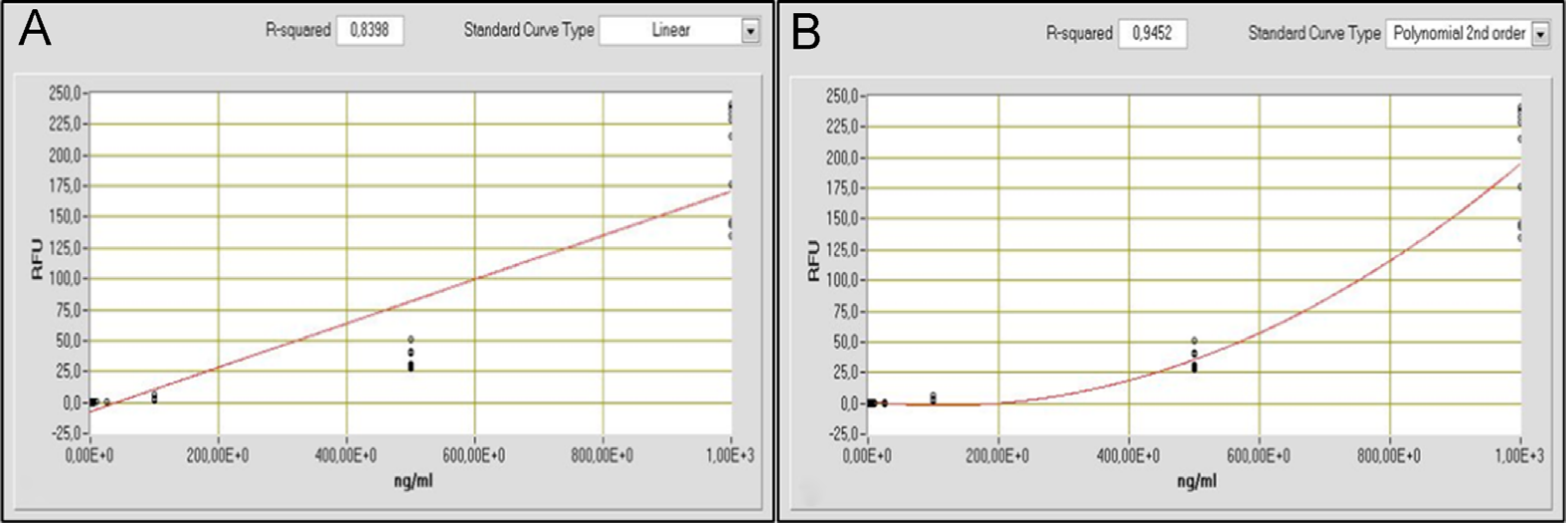
Mathematical adjustment of the standard curve data obtained for salmon sperm DNA (low molecular weight) using NanoDrop׳s software: A) Linear adjustment with R-squared 0.8398; B) 2nd order polynomial adjustment with R-squared 0.9452.

The dataset of this article provides information on the quantification of high molecular weight DNA, using λDNA solutions, versus fragmented DNA, using salmon sperm DNA. Fig. 1 shows the standard curve obtained using λDNA solutions, while Fig. 2 shows the standard curve for the same DNA concentration range (0–1000 ng mL^-1^) obtained using salmon sperm DNA, including the different tested adjustments for the data obtained.

Three mathematical models were developed and compared with the equipment׳s software, being evaluated in terms of accuracy and precision in order to find a curve that would fit better the standards data for this type of fragmented DNA. The evaluation of precision, in terms of repeatability of the DNA quantification, was performed by testing 10 different assays. The results obtained using the three mathematical models are detailed in Table 1. The model based on weighted least squares regression, allows the quantification of samples with concentrations down to 75 ng mL^-1^ with %RSD lower than 20% for concentrations from 75 to 300 ng mL^-1^ and lower than 10% for concentrations from 300 to 1000 ng mL^-1^. The least squares regression showed a %RSD lower than 30% and lower than 11%, while the weighted ridge regression showed a %RSD lower than 25% and lower than 10% for the same concentration ranges, respectively.

**Table 1:** Quantification results for salmon sperm DNA samples from the 10 experiments performed, using the standard curves obtained with salmon sperm DNA: results obtained from the mathematical models adjustment using the algorithm developed.

**Sample ID**	**Concentration (ng mL**^**-1**^**)**	**Average Concentration Measured ± Standard deviation error between measurements (ng mL**^**-1**^**)**											
		**ASSAY 1**						**ASSAY 2**					
		**Least Squares Regression**		**Weighted Least Squares Regression**		**Weighted Ridge Regression**		**Least Squares Regression**		**Weighted Least Squares Regression**		**Weighted Ridge Regression**	
**C1**	**1**	0.0	n.c.	0.0	n.c.	0.0	n.c.	54.8	n.c.	55.5	n.c.	54.9	n.c.
**C2**	**5**	11.6	± 9.6	13.9	± 8.3	10.2	± 4.2	52.2	± 53.5	53.4	± 51.4	53.6	± 50.6
**C3**	**10**	17.8	± 12.8	19.5	± 11.3	13.3	± 6.1	0.0	n.c.	0.0	n.c.	0.0	n.c.
**C4**	**25**	14.7	± 14.0	11.7	± 12.2	9.3	± 6.1	13.2	± 16.3	16.1	± 14.9	17.2	± 13.6
**C5**	**50**	39.6	± 14.9	39.3	± 13.8	26.5	± 10.5	52.3	± 25.3	53.2	± 24.3	53.0	± 24.0
**C6**	**75**	54.4	± 6.9	53.1	± 6.6	37.3	± 6.2	68.4	± 32.3	68.8	± 31.5	68.5	± 31.5
**C7**	**100**	73.6	± 12.4	71.6	± 12.1	60.0	± 18.3	83.7	± 16.1	83.6	± 15.7	83.2	± 15.8
**C8**	**200**	154.9	± 14.5	153.0	± 14.7	200.9	± 19.4	231.7	± 10.7	230.6	± 10.7	232.0	± 10.8
**C9**	**300**	243.9	± 8.9	243.3	± 9.0	293.6	± 7.8	299.2	± 13.0	298.0	± 13.0	299.9	± 13.1
**C10**	**400**	395.2	± 17.6	396.5	± 17.8	417.3	± 13.9	407.0	± 19.0	405.9	± 19.0	408.2	± 19.0
**C11**	**500**	512.2	± 35.6	514.0	± 35.7	509.8	± 28.4	475.5	± 30.7	474.6	± 30.8	476.9	± 30.7
**C12**	**600**	621.2	± 8.9	623.0	± 8.9	598.8	± 7.5	638.2	± 16.8	637.5	± 16.8	639.4	± 16.7
**C13**	**700**	761.1	± 11.2	762.5	± 11.2	722.9	± 10.5	719.6	± 26.0	719.1	± 26.0	720.6	± 25.9
**C14**	**800**	807.6	± 12.9	808.7	± 12.9	767.9	± 12.9	847.2	± 7.8	846.9	± 7.9	847.7	± 7.8
**C15**	**900**	910.7	± 11.8	911.1	± 11.8	879.2	± 14.1	923.7	± 17.7	923.6	± 17.7	923.9	± 17.6
**C16**	**1000**	983.7	± 16.1	983.6	± 15.9	975.5	± 23.9	967.7	± 38.5	967.7	± 38.6	967.7	± 38.3
**Sample ID**	**Concentration (ng mL**^**-1**^**)**	**ASSAY 3**						**ASSAY 4**					
		**Least Squares Regression**		**Weighted Least Squares Regression**		**Weighted Ridge Regression**		**Least Squares Regression**		**Weighted Least Squares Regression**		**Weighted Ridge Regression**	
**C1**	**1**	9.4	n.c.	18.5	n.c.	28.6	n.c.	0.0	n.c.	8.9	n.c.	5.1	n.c.
**C2**	**5**	2.7	n.c.	0.3	n.c.	0.0	n.c.	26.7	n.c.	23.6	n.c.	13.6	n.c.
**C3**	**10**	5.0	n.c.	0.3	n.c.	0.0	n.c.	74.2	± 29.6	65.6	± 30.0	65.6	± 50.8
**C4**	**25**	9.4	n.c.	18.5	n.c.	28.6	n.c.	20.8	± 17.8	21.0	± 9.9	12.2	± 6.3
**C5**	**50**	9.4	n.c.	18.5	n.c.	28.6	n.c.	55.5	± 24.2	48.0	± 20.4	38.7	± 22.2
**C6**	**75**	28.8	± 8.3	62.4	± 16.3	77.0	± 16.5	76.8	± 20.8	67.5	± 21.6	67.0	± 40.2
**C7**	**100**	62.6	± 20.0	108.4	± 22.1	120.9	± 20.6	83.2	± 10.4	73.5	± 10.9	73.9	± 21.6
**C8**	**200**	124.5	± 36.1	167.8	± 30.8	176.4	± 28.9	146.0	± 20.5	143.3	± 23.3	189.9	± 27.0
**C9**	**300**	253.4	± 22.4	274.6	± 18.7	277.7	± 18.0	276.5	± 18.9	289.0	± 20.4	321.2	± 15.8
**C10**	**400**	357.1	± 19.8	364.1	± 17.6	364.6	± 17.2	383.9	± 22.1	402.9	± 22.9	408.4	± 17.4
**C11**	**500**	453.6	± 11.1	452.7	± 10.5	451.8	± 10.4	459.9	± 25.7	480.7	± 25.9	468.2	± 20.2
**C12**	**600**	631.7	± 15.1	626.9	± 15.2	625.8	± 15.3	626.5	± 12.2	645.7	± 11.8	601.6	± 10.1
**C13**	**700**	744.8	± 28.9	742.4	± 29.7	742.4	± 30.1	703.5	± 24.8	720.0	± 23.7	667.3	± 21.8
**C14**	**800**	821.4	± 29.2	821.8	± 30.5	823.0	± 31.0	814.9	± 18.9	826.1	± 17.9	770.5	± 19.0
**C15**	**900**	914.0	± 9.7	918.9	± 10.2	921.8	± 10.4	933.5	± 25.7	937.4	± 24.0	902.8	± 32.3
**C16**	**1000**	962.8	± 27.0	970.5	± 28.6	974.6	± 29.3	973.0	± 30.5	974.1	± 28.4	959.2	± 49.0
**SampleID**	**Concentration(ng mL**^**-1**^**)**	**ASSAY 5**						**ASSAY 6**					
		**Least Squares Regression**		**Weighted Least Squares Regression**		**Weighted Ridge Regression**		**Least Squares Regression**		**Weighted Least Squares Regression**		**Weighted Ridge Regression**	
**C1**	**1**	0.0	n.c.	0.0	n.c.	0.0	n.c.	30.1	± 11.4	36.7	± 12.5	38.3	± 11.8
**C2**	**5**	20.5	± 10.1	18.3	± 10.4	18.5	± 10.6	15.1	± 11.5	20.0	± 13.5	21.7	± 14.8
**C3**	**10**	0.0	n.c.	0.0	n.c.	0.0	n.c.	0.0	n.c.	0.0	n.c.	0.0	n.c.
**C4**	**25**	24.4	± 13.0	22.3	± 13.4	22.5	± 13.6	6.9	n.c.	5.3	± 7.2	11.2	n.c.
**C5**	**50**	37.9	± 4.6	36.1	± 4.7	36.6	± 4.7	43.3	± 15.1	50.4	± 15.7	50.6	± 14.0
**C6**	**75**	59.1	± 6.8	57.7	± 6.9	58.3	± 6.9	89.4	± 3.2	96.7	± 3.2	90.8	± 2.8
**C7**	**100**	90.9	± 9.8	89.9	± 9.9	90.6	± 9.9	80.1	± 15.9	87.4	± 15.7	82.8	± 13.7
**C8**	**200**	156.5	± 6.8	156.2	± 6.9	157.0	± 6.9	176.4	± 11.2	181.7	± 11.0	167.4	± 10.3
**C9**	**300**	277.8	± 11.8	278.3	± 11.9	279.1	± 11.8	330.3	± 4.2	332.2	± 4.1	317.3	± 4.3
**C10**	**400**	395.6	± 15.1	396.6	± 15.2	397.3	± 15.1	434.5	± 11.3	434.8	± 11.2	428.3	± 12.3
**C11**	**500**	478.5	± 14.8	479.6	± 14.8	480.2	± 14.7	489.9	± 20.4	489.6	± 20.2	488.9	± 22.4
**C12**	**600**	550.7	± 24.4	551.9	± 24.4	552.2	± 24.3	631.0	± 5.5	630.0	± 5.5	643.0	± 5.9
**C13**	**700**	626.5	± 11.8	627.7	± 11.8	627.7	± 11.8	724.4	± 6.7	723.3	± 6.7	741.0	± 6.9
**C14**	**800**	771.5	± 11.9	772.3	± 11.9	771.7	± 11.8	858.2	± 12.4	857.7	± 12.5	872.5	± 11.6
**C15**	**900**	876.7	± 14.2	877.0	± 14.2	875.9	± 14.1	929.0	± 5.0	929.2	± 5.1	937.7	± 4.5
**C16**	**1000**	993.7	± 24.8	993.1	± 24.6	991.3	± 24.5	1009.1	± 27.5	1010.1	± 27.8	1007.6	± 23.3
**Sample ID**	**Concentration (ng mL**^**-1**^**)**	**Average Concentration Measured ± Standard deviation error between measurements (ng mL**^**-1**^**)**											
		**ASSAY 7**						**ASSAY 8**					
		**Least Squares Regression**		**Weighted Least Squares Regression**		**Weighted Ridge Regression**		**Least Squares Regression**		**Weighted Least Squares Regression**		**Weighted Ridge Regression**	
**C1**	**1**	61.3	± 19.0	59.9	± 19.3	56.4	± 19.7	80.7	n.c.	78.4	n.c.	76.2	n.c.
**C2**	**5**	43.4	n.c.	41.7	n.c.	26.1	± 16.6	0.0	n.c.	0.0	n.c.	0.0	n.c.
**C3**	**10**	0.0	n.c.	0.0	n.c.	0.0	± n.c.	0.0	n.c.	0.0	n.c.	60.1	n.c.
**C4**	**25**	51.7	n.c.	50.3	n.c.	30.5	± 22.7	0.0	n.c.	0.0	n.c.	0.0	n.c.
**C5**	**50**	48.8	± 12.6	47.2	± 13.0	43.5	± 13.1	0.0	n.c.	0.0	n.c.	60.1	n.c.
**C6**	**75**	75.8	± 13.3	74.7	± 13.4	71.5	± 13.7	94.7	± 7.8	93.3	± 8.2	94.3	± 9.7
**C7**	**100**	94.3	± 3.9	93.4	± 3.9	90.7	± 4.0	105.6	± 20.3	104.7	± 21.3	107.3	± 23.8
**C8**	**200**	154.5	± 10.5	154.0	± 10.5	152.8	± 10.8	195.6	± 5.7	198.5	± 5.9	207.5	± 6.0
**C9**	**300**	252.1	± 8.2	252.0	± 8.3	252.9	± 8.4	263.6	± 9.9	268.6	± 10.2	276.6	± 9.8
**C10**	**400**	339.5	± 9.3	339.7	± 9.3	342.1	± 9.4	374.9	± 4.8	382.4	± 4.9	383.9	± 4.5
**C11**	**500**	499.5	± 21.0	500.0	± 21.0	503.9	± 21.1	478.9	± 27.0	487.7	± 27.2	480.6	± 24.9
**C12**	**600**	570.9	± 10.7	571.5	± 10.7	575.5	± 10.7	556.4	± 4.9	565.4	± 4.9	551.8	± 4.5
**C13**	**700**	623.8	± 11.6	624.3	± 11.6	628.3	± 11.6	691.9	± 20.7	699.8	± 20.4	677.5	± 19.6
**C14**	**800**	773.0	± 16.2	773.5	± 16.2	776.1	± 16.0	817.4	± 5.5	822.8	± 5.3	798.7	± 5.5
**C15**	**900**	877.2	± 9.3	877.5	± 9.3	878.5	± 9.1	891.2	± 23.4	894.2	± 22.6	874.9	± 24.8
**C16**	**1000**	993.4	± 37.4	993.3	± 37.3	991.5	± 36.3	1001.2	± 45.8	999.5	± 43.6	1004.0	± 60.3
**Sample ID**	**Concentration (ng mL**^**-1**^**)**	**ASSAY 9**						**ASSAY 10**					
		**Least Squares Regression**		**Weighted Least Squares Regression**		**Weighted Ridge Regression**		**Least Squares Regression**		**Weighted Least Squares Regression**		**Weighted Ridge Regression**	
**C1**	**1**	35.0	n.c.	36.4	n.c.	26.6	n.c.	36.6	± 0.0	36.3	± 0.0	27.2	± 0.0
**C2**	**5**	46.4	± 6.8	46.5	± 6.2	39.4	± 7.8	56.7	± 24.3	56.0	± 23.1	50.1	± 24.0
**C3**	**10**	0.0	n.c.	0.0	n.c.	0.0	n.c.	1.7	n.c.	6.2	n.c.	3.4	± 2.6
**C4**	**25**	48.9	± 3.1	42.2	± 11.7	33.6	± 15.4	18.9	± 12.4	20.3	± 10.8	10.1	± 9.2
**C5**	**50**	56.3	± 8.4	55.9	± 8.1	51.0	± 9.8	33.3	± 11.2	33.3	± 10.4	25.1	± 9.2
**C6**	**75**	81.2	± 13.1	80.4	± 13.1	79.9	± 15.2	74.6	± 11.5	73.2	± 11.3	69.3	± 13.9
**C7**	**100**	89.8	± 6.4	88.9	± 6.4	89.9	± 7.3	89.5	± 10.8	88.0	± 10.8	88.2	± 14.0
**C8**	**200**	194.4	± 6.4	194.6	± 6.5	204.0	± 6.7	202.5	± 9.3	201.7	± 9.4	223.5	± 10.0
**C9**	**300**	241.0	± 7.9	241.8	± 8.0	251.9	± 8.0	299.4	± 6.8	299.5	± 6.8	320.2	± 6.4
**C10**	**400**	353.8	± 11.1	355.7	± 11.1	362.6	± 10.6	436.6	± 22.3	437.5	± 22.3	444.4	± 19.6
**C11**	**500**	491.3	± 9.8	493.6	± 9.8	491.9	± 9.1	509.6	± 41.4	510.5	± 41.3	508.4	± 36.2
**C12**	**600**	583.1	± 6.0	585.2	± 6.0	577.0	± 5.6	649.1	± 11.9	649.6	± 11.9	631.5	± 10.7
**C13**	**700**	616.6	± 18.7	618.4	± 18.5	608.1	± 17.4	705.7	± 6.7	705.8	± 6.6	682.9	± 6.2
**C14**	**800**	740.7	± 10.6	741.1	± 10.4	724.8	± 10.2	861.0	± 25.8	859.5	± 25.5	835.1	± 27.7
**C15**	**900**	893.5	± 32.2	890.6	± 31.3	878.3	± 34.0	928.7	± 4.6	926.3	± 4.5	909.6	± 5.4
**C16**	**1000**	972.3	± 21.4	967.0	± 20.7	966.4	± 25.6	976.3	± 27.3	973.2	± 26.9	969.2	± 36.0

n.c. – not calculated (the standard deviation error was not calculated when the model could only estimate less than two concentration values from the RFU measurements obtained for the sample)

**Fig. 3:**
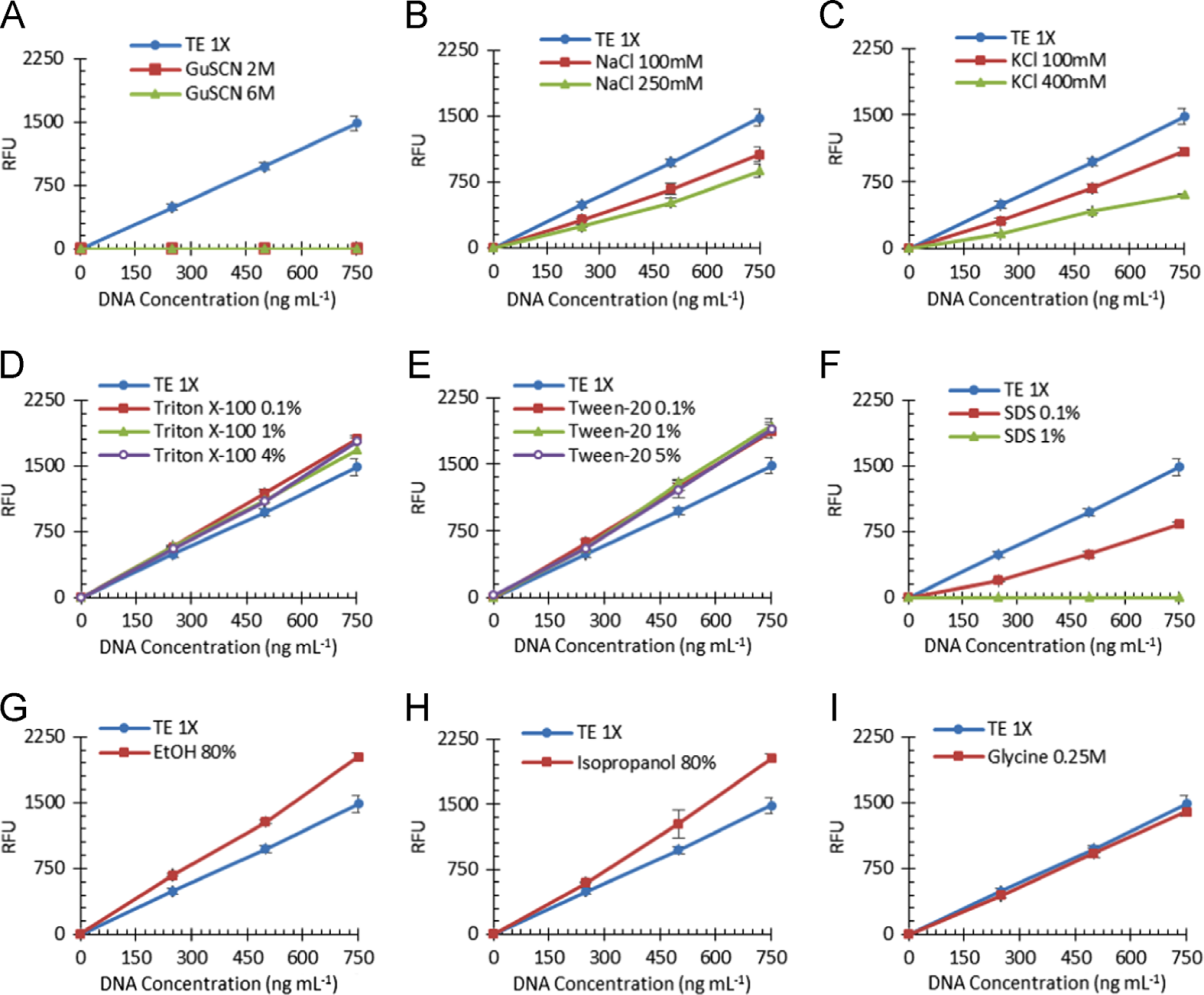
Influence of contaminants on the sensitivity of the PicoGreen DNA quantification assay using λDNA samples. Buffer TE 1x was used as a reference for comparison with other buffers containing: A) GuSCN 2 M and 6 M; B) NaCl 100 mM and 250 mM; C) KCl 100 mM and 400 mM; D) Triton X-100 0.1%, 1% and 4% (v/v); E) Tween-20 0.1%, 1% and 5% (v/v); F) SDS 0.1% and 1% (w/v); G) Ethanol 80% (v/v); H) Isopropanol 80% (v/v); I) Glycine 0.25 M.

In the present data article the influence of some compounds commonly used in µSPE-based methods on the accuracy of the quantification method was evaluated using λDNA solutions, which is a much larger DNA compared to the salmon sperm DNA tested and it is frequently used for the optimization of DNA purification devices. As represented in Fig. 3, the effect of each compound on the fluorescence signal was tested using different λDNA concentrations. The percent errors calculated for each condition are described in Table 2.

**Table 2:** Percent Errors calculated from the study of the influence of some compounds on the PicoGreen assay using λDNA samples.

**Compound**	**Concentration**	**Error**	**Compound**	**Concentration**	**Error**
**GuSCN**	2 M	− 99.9%	**Tween-20**	0.1% (v/v)	+ 26.1%
	6 M	− 99.9%		1% (v/v)	+ 25.1%
**NaCl**	100 mM	− 31.8%		5% (v/v)	+ 21.9%
	250 mM	− 45.8%	**SDS**	0.1% (w/v)	− 51.3%
**KCl**	100 mM	− 31.5%		1% (w/v)	− 99.7%
	400 mM	− 61.1%	**Ethanol**	80% (v/v)	+ 34.9%
**Triton X-100**	0.1% (v/v)	+ 20.3%	**Isopropanol**	80% (v/v)	+ 29.0%
	1% (v/v)	+ 15.1%	**Glycine**	0.25 M	− 6.9%
	4% (v/v)	+ 15.2%			

## 2 Experimental design, materials and methods

### 2.1 Experimental design

In this data article the influence of the DNA fragmentation level on the PicoGreen® fluorescence signal was evaluated by testing two types of DNA with different sizes: Bacteriophage λDNA (48502 bp) and low molecular weight salmon sperm DNA (≤ 300 bp). The standard curves required for DNA quantification were obtained using NanoDrop 3300 software and compared for both DNA types. Regarding the salmon sperm DNA, the three mathematical models described in the related research article [1] (least squares, weighted least squares and weighted ridge regressions) were implemented, being these curves compared with the one obtained using NanoDrop 3300 software. In order to evaluate the precision of these mathematical models under varied conditions, in terms of repeatability, a total of 10 assays were performed and the relative standard deviation (% RSD) was calculated as an indication of precision regarding variations from assay to assay.

The influence of some compounds commonly used in DNA extraction and purification protocols was also evaluated using λDNA solutions, which is a type of DNA commonly used for the optimization of microfluidic devices for DNA purification. Percent errors were calculated as an indication of effect of these compounds on the accuracy of the quantification method, in a sense of bias.

### 2.2 Materials

The data was obtained using bacteriophage λDNA (cIind 1 ts857 Sam7) (Alfagene®, Carcavelos, Portugal) and low molecular weight salmon sperm DNA (Sigma-Aldrich®, St. Louis, MO, US). The fluorescence signal of the different DNA solutions prepared was acquired using Quant-iT^TM^ PicoGreen® dsDNA Assay kit (Molecular probes Inc., Eugene, USA) in combination with NanoDrop 3300 (Thermo Scientific™, Waltham, MA, US). The influence of some compounds commonly used in DNA extraction and purification protocols was evaluated using solutions of Tris-Hydrochloride (Tris–HCl), ethylenediaminetetraacetic acid (EDTA), Tris-base, guanidine thiocyanate (GuSCN), glycine, sodium chloride (NaCl), potassium chloride (KCl), ethanol, isopropanol, Triton X-100, Tween-20 and sodium dodecyl sulfate (SDS) prepared with different concentrations, as described in Table 2.

### 2.3 DNA quantification method

The DNA quantification was performed using NanoDrop 3300 and PicoGreen® fluorescence. This quantification method requires a standard curve in order to correlate the emitted fluorescence with the dsDNA concentration of the samples. The standard curve was obtained by measuring the fluorescence signal of serially diluted dsDNA solutions with concentrations from 0 to 1000 ng mL^-1^ in buffer TE 1×. For each assay a fresh PicoGreen® working solution was prepared by mixing 5 µL of the dye stock with 995 µL of buffer TE 1×. The standard dilutions and the samples were mixed with the working solution in a volume ratio of 1:1 in a total of 20 µL. After 5 min, these solutions were measured to obtain the respective fluorescence signals.
